# The obesity paradox in varicocele – is the protective effect real?

**DOI:** 10.1590/S1677-5538.IBJU.2019.0210.1

**Published:** 2020-11-18

**Authors:** Sandro C. Esteves, Marcello S. Cocuzza

**Affiliations:** 1 ANDROFERT Andrology and Human Reproduction Clinic Campinas Brasil ANDROFERT, Andrology and Human Reproduction Clinic, Campinas, Brasil; 2 Universidade Estadual de Campinas Departamento de Cirurgia Divisão de Urologia CampinasSP Brasil Departamento de Cirurgia, Divisão de Urologia, Universidade Estadual de Campinas, UNICAMP, Campinas, SP, Brasil; 3 Aarhus University Faculty of Health Aarhus Denmark Faculty of Health, Aarhus University, Aarhus, Denmark; 4 Universidade de São Paulo Divisão de Urologia Centro de Reprodução Humana SP Brasil Centro de Reprodução Humana, Divisão de Urologia, Universidade de São Paulo, SP, Brasil

## COMMENT

Varicocele is a common vascular abnormality resulting from the enlargement of the pampiniform venous plexus (1). The condition is found in 15-20% of the male adult population and increases the infertility risk (2, 3). Up to 40% of men complaining of infertility have a varicocele detected during investigation. Its urological relevance relates primarily to its potential treatment by surgery, which may restore or improve fertility, thus allowing couples to achieve natural conception or increased success rates when using assisted conception (4-7).

The classical teaching is that varicocele is more common in young men who are taller and thinner. Studies looking at body habitus and varicocele seem to indicate that the condition is more common in men with lower body mass index (BMI). Some evidence also indicates that the lower prevalence of varicocele in obese men is independent of physical examination due to the inverse relationship between BMI and varicocele diagnosed by ultrasound (8). However, there is still a large cohort of overweight/obese men who suffer from this condition (8).

A recent systematic review and meta-analysis investigated the association between BMI and varicocele. In their study, Xiao-Bin and co-workers (9) summarized the data of eleven case-control and cross-sectional studies, including over one million men, and concluded that being overweight or obese lower the varicocele risk, whereas underweight increases it. The decreased risk of having a varicocele was evident and consistent among obese men; however, the effect was more equivocal among overweight men as in five of the included studies, the odds ratio 95% confidence interval crossed 1. By contrast, there was an increased risk of varicocele among underweight men, although the largest study included in the authors meta-analysis failed to confirm the relationship.

The authors discussed two possible theories to explain their findings. First, the ‘protective’ effect of adipose tissue deposited between the aorta and the superior mesenteric artery, which would avoid the ‘nutcracker’ phenomenon. Second, the operator bias related to varicocele diagnosis by physical examination. Although the authors favor the first hypothesis, it remains to be elucidated whether the excess retroperitoneal fat tissue would indeed deposit in that spot and confer protection. Noteworthy, one study evaluating spermatic vein diameter (SVD) reported a positive association between left spermatic vein diameter and BMI when the examinations were carried out in the supine position. The authors speculated that the increase in abdominal pressure in supine could be related to central fat deposition.8 Along these lines, although the real prevalence of varicocele caused by the nutcracker phenomenon is unknown (10), it is unlikely to be too frequent or even counterpartyed by adipose tissue location; otherwise, the recurrence rates after the gold-standard microsurgical varicocele repair would be much higher than reported (11).

On the other hand, what every urologist with expertise in male infertility does know is that obesity may affect the ability to make the varicocele diagnosis accurately using physical examination alone. Not only that, but there is a remarkable inter-operator variation in varicocele diagnosis by physical examination (12). In a recent study involving 78 patients, we found that the specificity and positive predictive value of physical examination were higher among experienced (male infertility experts) than in-training urologists (82.0% and 81.1% versus 67.2% and 70.6%). Moreover, agreements on varicocele diagnosis (k: 0.625 versus 0.517) and grading (k: 0.548 versus 0.418) by physical examination were higher among experienced than non-experienced urologists. Our findings underline the limitations of physical examination on varicocele diagnosis. Thus, we advocate that physical examination should be followed by CDU to decrease the number of false positives and increase the diagnostic accuracy of varicocele diagnosis, as recommended by the European Association of Urology male infertility guidelines. We feel the clinical utility of the latter is paramount during the work-up of the obese infertile men.

Xiao-Bin and co-workers correctly caution that their findings concern an association rather than a causal relationship. Their meta-analysis did not account for critical confounders such as operator-dependent diagnosis expertise, patient selection criteria, and whether varicocele was diagnosed by physical examination, Doppler ultrasound, or both. These factors are important confounders to control for, as in some studies the association between varicocele and BMI was not confirmed (13). Therefore, their findings may be attributed to the difficulties in performing PE for varicocele diagnosis in obese and underweight men.

Lastly, obesity is a disease that plagues modern society, making it unlikely that it may confer protection for any medical condition (14). With regards to obesity and male reproductive health, mounting evidence supports the notion that obesity has an adverse impact on male infertility, via a variety of pathophysiologic mechanisms, including HPG axis changes, adipokines, inflammation and oxidative stress, increased scrotal temperature, as well as genetic and epigenetic alterations (15). These effects ultimately result in abnormalities on conventional and advanced semen parameters, such as sperm DNA fragmentation (16, 17). Therefore, it is sound to consider that the adverse effect of obesity on male fertility can easily offset any arguably protective effect of obesity on varicocele risk.
